# The Mechanisms of Action of Triindolylmethane Derivatives on Lipid Membranes

**DOI:** 10.32607/20758251-2019-11-3-38-45

**Published:** 2019

**Authors:** S. S. Efimova, T. E. Tertychnaya, S. N. Lavrenov, O. S. Ostroumova

**Affiliations:** Institute of Cytology, Russian Academy of Sciences, Tikhoretsky Ave. 4, St. Petersburg, 194064, Russia; Gause Institute of New Antibiotics, Russian Academy of Medical Sciences, Bolshaya Pirogovskaya Str. 11, Moscow, 119021, Russia

**Keywords:** antimicrobial agents, antibiotics, turbomycin A, lipid bilayers, liposomes, ion-permeable nanopores

## Abstract

The effects of new synthetic antibacterial agents – tris(1-pentyl-1H-indol-3-yl)methylium chloride (LCTA-1975) and (1-(4-(dimethylamino)-2,5-dioxo-2,5-dihydro-1H-pyrrol-3-yl)-1H-indol-3-yl)bis(1-propyl- 1H-indol-3-yl)methylium chloride (LCTA-2701 – on model lipid membranes were studied. The ability of the tested agents to form ion-conductive transmembrane pores, influence the electrical stability of lipid bilayers and the phase transition of membrane lipids, and cause the deformation and fusion of lipid vesicles was investigated. It was established that both compounds exert a strong detergent effect on model membranes. The results of differential scanning microcalorimetry and measuring of the threshold transmembrane voltage that caused membrane breakdown before and after adsorption of LCTA-1975 and LCTA-2701 indicated that both agents cause disordering of membrane lipids. Synergism of the uncoupling action of antibiotics and the alkaloid capsaicin on model lipid membranes was shown. The threshold concentration of the antibiotic that caused an increase in the ion permeability of the lipid bilayer depended on the membrane lipid composition. It was lower by an order of magnitude in the case of negatively charged lipid bilayers than for the uncharged membranes. This can be explained by the positive charge of the tested agents. At the same time, LCTA-2701 was characterized by greater efficiency than LCTA-1975. In addition to its detergent action, LCTA-2701 can induce ion-permeable transmembrane pores: step-like current fluctuations corresponding to the opening and closing of individual ion channels were observed. The difference in the mechanisms of action might be related to the structural features of the antibiotic molecules: in the LCTA-1975 molecule, all three substituents at the nitrogen atoms of the indole rings are identical and represent n-alkyl (pentyl) groups, while LCTA-2701 contains a maleimide group, along with two alkyl substituents (n-propyl). The obtained results might be relevant to our understanding of the mechanism of action of new antibacterial agents, explaining the difference in the selectivity of action of the tested agents on the target microorganisms and their toxicity to human cells. Model lipid membranes should be used in further studies of the trends in the modification and improvement of the structures of new antibacterial agents.

## INTRODUCTION

The main challenge of modern antibiotic therapy is the side effects of antibacterial agents and the growing resistance of pathogenic bacteria to them, which results in the loss of their clinical potency by a number of drugs. One of the promising ways to overcoming these difficulties is to modify natural antibiotic compounds in order to create semisynthetic derivatives, which not only exhibit pronounced activity against resistant microorganisms, but also exert an extended spectrum of action, compared to their original versions.

The antibiotic turbomycin A, first isolated as a metabolic product of Saccharomyces cerevisiae, exhibits relatively low activity against Gram-positive bacteria [1] and presents a salt of tris(indol-3-yl)methylium [2]. Introduction of alkyl substituents at nitrogen atoms of the indole rings of the antibiotic was shown to significantly increase the antibacterial effect, expand the antibacterial spectrum, and induce the antitumor activity of the drug [3, 4].

Of great interest are the novel homologs of turbomycin A, namely, tris(1-pentyl-1H-indol-3-yl) methylium chloride (LCTA-1975) and (1-(4-(dimethylamino)- 2,5-dioxo-2.5-dihydro-1H-pyrrol-3-yl)-1Hindol- 3-yl)bis(1-propyl-1H-indol-3-yl)methylium chloride (LCTA-2701), which were synthesized at the Gause Institute of New Antibiotics. In addition to its high antibacterial activity against multidrug-resistant Gram-positive bacteria, LCTA-1975 also induces the apoptosis of tumor cells via the NF-kB signaling pathway [3, 5, 6]. LCTA-2701, which exhibits approximately the same level of antibacterial activity as LCTA-1975, is significantly less toxic to human cells (donor fibroblasts) [7]. This work presents a study of the mechanisms of interaction of LCTA-1975 and LCTA-2701 with lipid bilayers, including those that simulate the membranes of target cells.

## EXPERIMENTAL

**Fig. 1:**
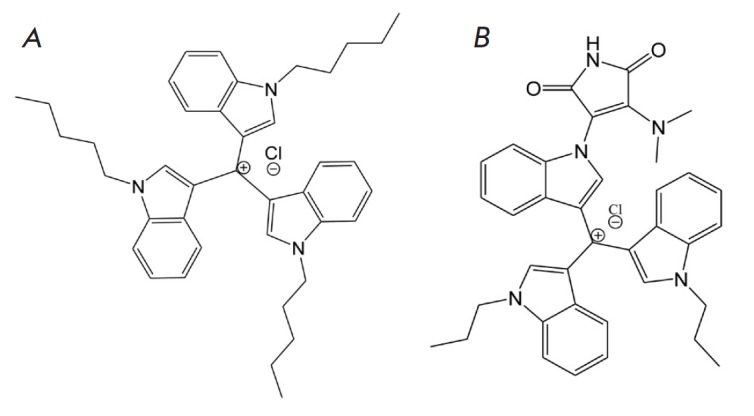
The chemical structure of triindolylmethane derivatives: LCTA-1975 (A) and LCTA-2701 (B)

The following compounds were used in the study: 1,2-dioleoyl-sn-glycero-3-phospho-L-serine (DOPS), 1,2-dioleoyl-sn-glycero-3-phosphoethanolamine (DOPE), 1,2-diphytanoyl-sn-glycero-3-phosphocholine (DPhPC), 1-palmitoyl-2-oleoyl-sn-glycero-3-phosphocholine (POPC), and 1,2-dipalmitoyl-sn-glycero- 3-phosphocholine (DPPC) (Avanti Polar Lipids, USA); KCl, KOH, HEPES, pentane, ethanol, hexadecane, dimethyl sulfoxide, capsaicin, caffeine (Sigma, USA). The chemical structures of the triindolylmethane derivatives LCTA-1975 and LCTA-2701 synthesized at the Gause Institute of New Antibiotics are presented in Fig. 1.

**Preparation of planar lipid bilayers and recording of the transmembrane currents**

Bilayer lipid membranes were formed according to the Montal and Müller technique [8], by mixing condensed lipid monolayers over a hole in a Teflon film dividing the experimental chamber into two (the cis and trans) compartments. The volume of each compartment was 1.5 ml, the thickness of the Teflon film was 10 μm, and the hole diameter was about 50 μm. The hole in the Teflon film was treated with hexadecane prior to membrane formation. Planar lipid bilayers were formed from DPhPC or POPC, as well as from an equimolar mixture of DOPS and DOPE. The experiments were carried out using aqueous electrolyte solutions (0.1 M KCl, pH 7.4) with an identical ionic composition separated by a membrane. The acidity of the solutions (pH 7.4) was maintained using a 5 mM HEPES/KOH buffer.

The tested substances, LCTA-1975 and LCTA-2701, were added from a 10 mg/ml solution in dimethyl sulfoxide and H_2_O (1 : 1) to the cis compartment of the chamber to the final concentration as indicated in Table 1. At least four independent experiments (repeats) were performed for each agent-lipid bilayer system.

**Table 1:** The dependence between the membrane activity and concentration of triindolylmethane derivatives in the membrane-bathing solutions (C, μM)

Activity type	LCTA-1975			LCTA-2701		
	DOPS : DOPE (50 : 50 mol%)	DPhPC	POPC	DOPS : DOPE (50 : 50 mol%)	DPhPC	POPS
No activity	< 15	< 315	< 200	< 8	< 55	< 30
Ion-permeable pores	–	–	–	8-25	55-135	30-130
Detergent effect	> 15	> 315	> 200	> 25	> 135	> 130

*Note:*The concentration error is ≤ 10%.

Silver/silver chloride electrodes connected to the chamber solutions through 1.5% agarose bridges containing 2 M KCl were used to apply the transmembrane potential and record the transmembrane current. The potential of the current flow of cations from the cis to the trans compartment of the chamber was considered positive.

Transmembrane currents were measured and digitized in the voltage clamp mode using an Axopatch 200B and Digidata 1440A systems (Axon Instruments, USA). The data were processed using an eight-pole Bessel filter (Model 9002, Frequency Devices) at a filtering frequency of 1 kHz.

Transmembrane current recordings were processed using the Clampfit 9.0 software package (Axon Instruments). Statistical analysis of the data was performed using the Origin 8.0 software (Origin Lab., USA).

The conductance of the pore was determined as the ratio of the current flowing through a single pore to the transmembrane voltage. Histograms of current fluctuations were obtained for the values of the transmembrane currents determined by the changes in the current amplitude upon opening or closing of individual channels. Records of current fluctuations through membranes with a single integrated channel were used to determine the channel lifetime (the open state time). For each of the experimental systems, the conductance and pore lifetime values were presented as an arithmetic mean and a standard error (mean ± SE).

The threshold values of the transmembrane voltage Vbd, which causes the destruction of the DPhPC membranes before and after the addition of triindolylmethane derivatives to the membrane-bathing solutions, were measured by applying a voltage in the range of 0 to ± Vbd to the membrane. No differences were found between the positive and negative transmembrane potentials.

**Determination of changes in the electric potential at the membrane/aqueous solution interface upon introduction of the test derivatives**

The nonactin ionophore was added to the bathing solutions on both sides of the membrane to a final concentration of 10-7–10-6 M. Lipid membranes were formed from DPhPC in 0.1 M KCl, 5 mM HEPES/KOH buffer, pH 7.4, according to the procedure described above.

Bilayer conductance (G) was determined as the ratio of the steady-state transmembrane current to the transmembrane potential, which equaled 50 mV. Changes in the electric potential at the membrane/ aqueous solution interface caused by the introduction of the test derivatives (Δφ_d_) were determined as

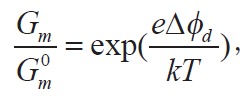



where G0 m and Gm are the values of the steady-state K+ conductance of the bilayer induced by nonactin before and after the introduction of the test agent.

The studied compounds LCTA-1975 and LCTA-2701 were added to the cis compartment of the experimental chamber from a 1 : 1 dimethyl sulfoxide-to-H_2_O solution to a final concentration of 300 and 150 μM, respectively.

**Confocal fluorescence microscopy of liposomesa**

Giant unilamellar liposomes were formed from POPC in an electric field using a commercial Nanion vesicle prep pro setup (Germany) on a pair of glasses coated with a conductive mixture of indium oxide and tin oxide with a standard protocol (alternating voltage with an amplitude of 3 V, 10 Hz frequency, 1 h, 25°C) according to [9].

The resulting suspension of liposomes was divided into aliquots. Triindolylmethane derivatives were added into the experimental samples at a lipid-to-agent ratio of 10 : 1. Aliquots were equilibrated for 30 min at room temperature, and 10 μl of the obtained liposome suspension was placed between the slide and cover glasses. Liposomes were observed in transmitted light on an Olympus FV3000 confocal microscope (Germany). Independent experiments (3 to 5) were carried out, and the average liposome diameter was determined for each of the experimental systems (mean ± SE).

**Differential scanning microcalorimetry of liposomes**

Giant unilamellar liposomes were formed from DPPC in an electric field as described above. The resulting liposome suspension was adjusted to 800 μl with a buffer solution (5 mM HEPES, pH 7.4). The final lipid concentration was 5 mM. LCTA-1975 and LCTA-2701 were introduced into the experimental samples at a lipid-to-agent ratio of 10 : 1 or 5 : 1. The control samples remained unmodified. Thermograms of liposome suspensions were obtained using a μDSC7 differential scanning microcalorimeter (Setaram, France). The required amount of suspension was placed in a cell and heated at a constant rate of 0.2 K/min; an equivalent volume of the buffer solution was placed in the second cell. The reproducibility of the temperature depend ence of the heat capacity was achieved by reheating the sample immediately after cooling. Thermogram peaks were characterized by the temperatures of the pre-transition (T_p_) and the main transition (T_m_), and the width at half-maximum of the main peak (T_1/2_), which characterizes the cooperativity of the transition of DPPC from gel phase to liquid phase, as well as the change in the enthalpy of the main phase transition (ΔH).

## RESULTS AND DISCUSSION

**Fig. 2:**
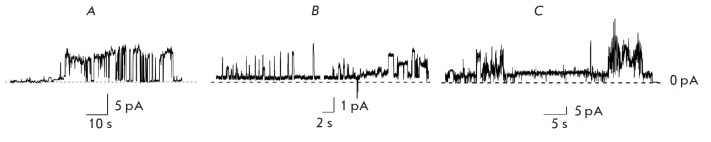
Current fluctuations corresponding to the opening and closing of individual pores induced by LCTA-2701 in the planar lipid bilayer at antibiotic concentrations of 10 (A), 70 (B), and 100 (C) μM. Membranes were formed from the DOPS:DOPE (50 : 50 mol%) (A), DPhPC (B) and POPC (C) and bathed in 0.1 M KCl, 5 mM HEPES, pH 7.4. The transmembrane voltage was equal to 100 mV

The membrane activity of the synthetic homologs of turbomycin A, namely LCTA-1975 and LCTA-2701, has been studied. Table 1 presents the characteristics of the action of the tested agents on lipid bilayers. Introduction of LCTA-1975 to membranes made from an equimolar mixture of DOPS and DOPE to a final concentration of 15 μM does not cause any noticeable fluctuations in the transmembrane current. An increase in the agent concentration disturbs the bilayer, with its subsequent breakdown. Addition of LCTA-2701 to a concentration of 8 μM does not affect the ionic permeability of negatively charged membranes. Introduction of LCTA-2701 to a concentration of 8–25 μM into the cis compartment solution increases the membrane conductance through the formation of ion-permeable pores. Figure 2A shows examples of recordings of step-like current fluctuations for DOPS : DOPE (50 : 50 mol%) membranes in the presence of LCTA- 2701. It can be seen from Fig. 2A that the pores have differing conductance. Pore conductance varies from 5 to 100 pS, and their lifetime ranges from 0.1 to 5 s. A further increase in LCTA-2701 concentration results in the disintegration of the lipid bilayer.

Unlike for negatively charged membranes, including DOPS, the tested agents exert a detergent effect with respect to the bilayers composed of neutral lipids, DPhPC or POPC, at concentrations higher by an order of magnitude (Table 1). An increase in the concentrations of LCTA-1975 and LCTA-2701 to 200 and 130 μM, respectively, disturbs the stability of uncharged membranes and causes their breakdown. LCTA-2701 at a concentration of 30–130 μM also demonstrates pore-forming ability. Recordings of current fluctuations corresponding to the opening and closing of the transmembrane pores induced by this substance in the DPhPC or POPC bilayer are shown in Fig. 2B,C.

The obtained results allow us to conclude that the test compounds act differently on the model lipid membranes: both agents exhibit detergent activity, while LCTA-2701 is also capable of inducing transmembrane pores. It should be noted that the type of membrane activity for these substances does not depend on the membrane composition. The threshold concentration at which the destructive effect of the tested agents is manifested is determined by the surface charge of the bilayer. A possible explanation for this may be the positive charge of the tested compounds, which contributes to their sorption on the negatively charged membranes composed of DOPS and DOPE. In addition, the membrane activity of the tested derivatives is virtually independent of the shape of the membrane-forming lipids. The effect of the tested substances on the membranes of cone-shaped DPhPC and cylindrical POPC molecules [10, 11] manifests itself at similar concentrations. The threshold concentration that causes an increase in the ionic permeability of the lipid bilayer also depends on the type of agent. LCTA-2701 is more effective than LCTA-1975 with respect to both negatively charged and neutral membranes.

The planar lipid bilayers formed from DPhPC exhibit the highest electrical stability in the absence of any modifiers. The threshold value of the transmembrane voltage (Vbd) that causes a disruption of DPhPC membranes is 450 ± 30 mV. Introduction of LCTA-1975 or LCTA-2701 to a concentration of 100 μM leads to a drop in Vbd to 310 ± 30 mV and 370 ± 30 mV, respectively. This indicates that the electrical stability of the membrane decreases in the presence of these substances. The results suggest that the tested agents exhibit a disordering effect on the lipids in the bilayer.

The proposed assumption is independently confirmed by the results of a study focused on the effect of alkaloids on the membrane activity of LCTA-1975 or LCTA-2701. Comprehensive studies using such methods as differential scanning microcalorimetry, X-ray diffraction, fluorescence probe spectroscopy, and NMR demonstrated the significant effect of capsaicin, an alkaloid from chili pepper, on the phase transitions of membrane lipids [12, 13]. Capsaicin significantly reduces the melting temperature and cooperativity of dimyristoylphosphatidylcholine [14] and dipalmitoylphosphocholine [12]. Significant deconvolution of the peak corresponding to the main phase transition is observed in the thermogram at relatively high concentrations of the alkaloid, which is an indication that several mixed alkaloid–lipid phases coexist. Moreover, capsaicin enhances the ability of phosphoethanolamine to form non-lamellar inverted hexagonal phases. Capsaicin adsorption is believed to increase the negative spontaneous curvature of lipid monolayers [12, 15]. In palmitoyl–oleoyl–phosphocholine membranes, capsaicin is located between the lipid–water interphase and the plane of the double bond of the unsaturated acyl lipid chain [13]. A decrease in membrane stiffness in the presence of capsaicin was found to be responsible for the modulation of ion currents induced by the antibiotic gramicidin A [16, 17]. According to the reported data on the disordering effect of capsaicin, one can expect that the tested compounds would exhibit an enhanced detergent action in its presence. Indeed, the introduction of 0.4 mM capsaicin into the solutions bathing the DOPS : DOPE (50 : 50 mol%) membranes modified by LCTA-1975 or LCTA-2701 reduces the concentrations of the substances by 20–30%, thus causing a destabilization of the bilayer.

**Table 2:** Thermodynamic characteristics of DPPC liposomes in the absence and presence of triindolylmethane derivatives

Experimental system	Lipid: agent ratio	Peak No.	T_m_, °C	T_1/2_, °C	ΔH, kcal/mol
Control	–	1	41.2	0.6	13.3
LCTA-1975	10 : 1	1	41.0	0.9	12.3
		2	38.7		
		3	35.8		
		4	33.7		
	5 : 1	1	41.0	0.9	11.2
		2	37.8		
		3	35.5		
		4	34.4		
LCTA-2701	10 : 1	1	41/1	2.4	10.6
		2	39.1		
	5 : 1	1	41.2	1.8	9.6
		2	39.1		
		3	37.6		

*Note:*Tm is the temperature at the local maximum of heat capacity; T1/2 is the width at half -maximum of the main peak; ΔH is the enthalpy change of the main phase transition.

Interaction of another plant alkaloid, caffeine, with water molecules bound to neighboring lipids leads to a local increase in hydration and membrane thickness, while reducing its fluidity [18]. These results are consistent with calorimetry and molecular dynamics data according to which caffeine significantly compensates for the uncoupling effect of the local anesthetic tetracaine [19]. The effect is absent in the case of LCTA- 1975 and LCTA-2701. The non-specific interaction of caffeine with DOPS : DOPE (50 : 50 mol%) bilayers has almost no effect on the membrane activity of the tested substances. The inability of caffeine to compensate for the uncoupling effect of LCTA-1975 or LCTA-2701 may indicate a significant difference between the localization of these substances and caffeine in the membrane. According to [18, 20], xanthine molecules are located at the boundary between the regions of lipid “heads” and “tails.” LCTA molecules are more likely to be able to immerse in the hydrophobic region of the membrane due to their alkyl substituents, which results in an increase in the lateral pressure in this region and its expansion. The proximity of the location of the tested antibacterial agents and capsaicin in the membrane may explain the synergism of their disordering effect.

**Fig. 3:**
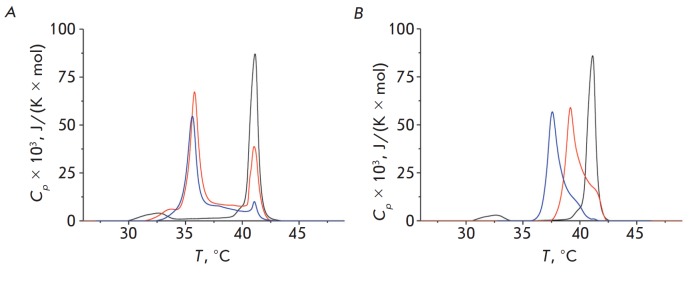
The thermograms of DPPC melting in the absence (control, black line) and presence of LCTA-1975 (A) and LCTA-2701 (B). The lipid : antibiotic ratio was equal to 10 : 1 *(red lines)* and 5 : 1 *(blue lines)*

**Fig. 4:**
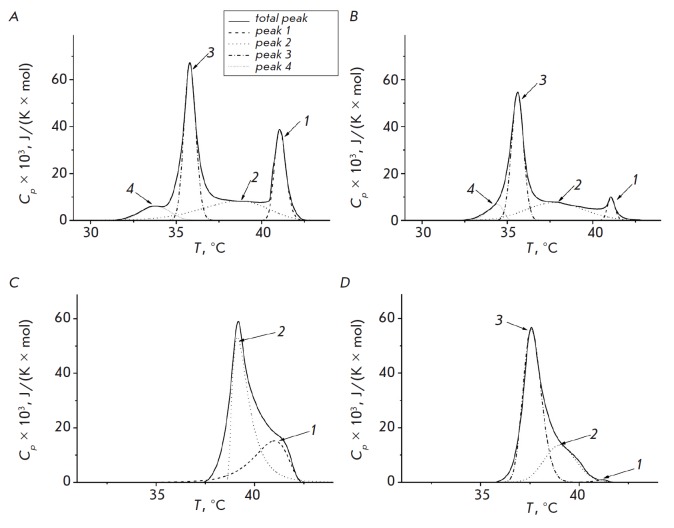
Deconvolution analysis of the main transition peak of DPPC in the presence of LCTA-1975 (A, B) and LCTA- 2701 (C, D). The lipid : antibiotic ratio was equal to 10 : 1 (A, C) and 5 : 1 (B, D). Peak parameters are presented in Table 2.

Figure 3 presents the thermograms for the DPPC liposomes in the absence (control, black lines) and in the presence of LCTA-1975 or LCTA-2701 in the suspension at the lipid-to-agent molar ratio of 10 : 1 (red lines) and 5 : 1 (blue lines). In the absence of LCTA derivatives, the pre-transition temperature Tp is 32.6°C; the temperature of the main phase transition Tm is 41.2°C; and the width at half-maximum of the main peak T_1/2_, which characterizes the cooperativity of the transition of DPPC from gel phase to liquid phase, does not exceed 0.6°C. Both tested agents significantly affect the DPPC melting process. In both cases, pre-transition is eliminated. Table 2 presents the Tm and T1/2 values for the lipid-to-agent ratio used in this study. Deconvolution of the peak corresponding to the main phase transition of DPPC should be noted, since its degree depends on the lipid-to-agent ratio observed in the presence of the tested compounds (Fig. 3). Figure 4 shows the result of deconvolution of the peak corresponding to the main phase transition of DPPC into including both DPPC and LCTA (peak 2 and(or) 3 in Fig. 4A–D). The drop in the temperature and cooperativity of DPPC transition in the presence of test substances can be associated with their immersion in the hydrophobic region of the bilayer, resulting in an increase in the area per lipid molecule and, consequently, an increase in the mobility of the lipid acyl tails. The obtained results also demonstrate that an increase in the LCTA-1975 and LCTA-2701 concentrations leads to a decrease in the change of the main phase transition enthalpy (ΔH): an approximately 10% decrease in ΔH is observed with a 2-fold increase in concentration. A decrease in ΔH can be due to the transition of part of the lipid to the non-lamellar phase [21-23]. In particular, the appearance of a pronounced peak at 34°C after the introduction of LCTA-1975 at all tested concentrations may indicate a significant change in the distribution of the lateral pressure in the membrane and appearance of non-layer lipid formations in the presence of this agent (peak 4 in Fig. 4A,B). separate components in the presence of the tested agents. The presence of several peaks indicates the coexistence of different phases. The number 1 peak in the thermograms (Fig. 4A–D) can be associated with the melting of pure DPPC, while the two remaining peaks are associated with the presence of different phases, including both DPPC and LCTA (peak 2 and(or) 3 in Fig. 4A–D). The drop in the temperature and cooperativity of DPPC transition in the presence of test substances can be associated with their immersion in the hydrophobic region of the bilayer, resulting in an increase in the area per lipid molecule and, consequently, an increase in the mobility of the lipid acyl tails. The obtained results also demonstrate that an increase in the LCTA-1975 and LCTA-2701 concentrations leads to a decrease in the change of the main phase transition enthalpy (ΔH): an approximately 10% decrease in ΔH is observed with a 2-fold increase in concentration. A decrease in ΔH can be due to the transition of part of the lipid to the non-lamellar phase [21, 22, 23]. In particular, the appearance of a pronounced peak at 34°C after the introduction of LCTA-1975 at all tested concentrations may indicate a significant change in the distribution of the lateral pressure in the membrane and appearance of non-layer lipid formations in the presence of this agent (peak 4 in Fig. 4A,B).

The effect of antibacterial substances on the boundary potential of the planar lipid bilayers formed of DOPS : DOPE (50 : 50 mol%) has been also studied. LCTA-1975 and LCTA-2701 do not affect the steadystate transmembrane current induced by the complex of nonactin ionophore with the potassium ion (Δφ_d_ = 1 ± 1 mV). This indicates the invariance of the distribution of the electric potential at the membrane/ aqueous solution interface during adsorption of the tested compounds.

POPC vesicles were studied using confocal microscopy before and after the introduction of LCTA derivatives into the suspension. The addition of LCTA-1975 or LCTA-2701 to POPC liposomes at a lipid-to-agent ratio of 10 : 1 does not change the spherical shape of lipid vesicles. The identical average liposome diameter before (15 ± 6 μm) and after (15 ± 7 μm) addition indicates that the tested agents do not cause fusion or division of the lipid vesicles.

## CONCLUSION

It has been established that the tested compounds act differently on model lipid membranes: LCTA-1975 demonstrates detergent properties, while LCTA-2701, in addition to its detergent activity, is also capable of inducing pores in phospholipid membranes. The differences in their mechanisms of action are due to their structural features: all three substituents at the nitrogen atoms of the indole rings in LCTA-1975 are identical and present n-alkyl (pentyl) groups, while LCTA-2701 contains, along with the two alkyl substituents (n-propyl), a maleimide group. The obtained results might be relevant to our understanding of the mechanism of action of new antibacterial agents, explaining the difference in the selectivity of their action on microorganisms and their cytotoxicity to human cells. Model lipid membranes should be used in further studies on the trends in the modification and improvement of the structures of new antibacterial agents.

## Abbreviations

LCTA-1975: tris(1-pentyl-1H-indol-3-yl)methylium chloride
LCTA-2701: (1-(4-(dimethylamino)-2,5-dioxo-2,5-dihydro-1H-pyrrol-3-yl)-1H-indol-3-yl)bis(1-propyl-1H-indol-3-yl)methylium chloride
DOPS: 1,2-dioleoyl-sn-glycero-3-phospho-L-serine
DOPE: 1,2-dioleoyl-sn-glycero-3-phosphoethanolamine
DPhPC: 1,2-diphytanoyl-sn-glycero-3-phosphocholine
POPC: 1-palmitoyl-2-oleoyl-sn-glycero-3-phosphocholine
DPPC: 1,2-dipalmitoyl-sn-glycero-3-phosphocholine

## References

[1] Palchaudhuri R., Nesterenko V., Hergenrother P.J. (2008). J. Am. Chem. Soc..

[2] Budzikiwicz H., Eckau H., Ehrenberg M. (1972). Tetrahedron Lett..

[3] Lavrenov S.N., Luzikov Y.N., Bykov E.E., Reznikova M.I., Stepanova E.V., Glazunova V.A., Volodina Y.L., Tatarsky V.V., Shtil A.A., Preobrazhenskaya M.N. (2010). Bioorg. Med. Chem..

[4] Stepanova E.V., Shtil’ A.A., Lavrenov S.N., Bukhman V.M., Inshakov A.N., Mirchink E.P., Trenin A.S., Preobrazhenskaya M.N. (2010). Russ. Chem. Bull..

[5] Isakova E.B., Treshchalin I..D.., Bodyagin D.A., Lavrenov S.N., Preobrazhenskaya M.N., Pereverzeva E.R. (2012). Russian Biotherapeutic Journal..

[6] Solomko E.Sh., Lavrenov S.N., Inshakov A.N., Abramov M.E., Preobrazhenskaya M.N., Stepanova E.V. (2012). Sarcomas of bones, soft tissues and skin tumors..

[7] Lavrenov S.N., Simonov A.Yu., Panov A.A., Lakatosh S.A., Isakova Ye.B., Tsvigun Ye.A., Bychkova O.P., Tatarsky V.V., Ivanova E.S., Mirchink E.P., Korolev A.M., Trenin A.S. (2018). Antibiotics and chemotherapy..

[8] Montal M., Mueller P. (1972). Proc. Natl. Acad. Sci. USA..

[9] Efimova S.S., Ostroumova O.S. (2017). Acta Naturae..

[10] Bezrukov S.M. (2000). Curr. Opin. Colloid. Interface Sci..

[11] Sakuma Y., Taniguchi T., Imai M. (2010). Biophys. J..

[12] Aranda F.J., Villalaín J., Gómez-Fernández J.C. (1995). Biochim. Biophys. Acta..

[13] Torrecillas A., Schneider M., Fernández-Martínez A.M., Ausili A., de Godos A.M., Corbalán-García S., Gómez-Fernández J.C. (2015). ACS Chem. Neurosci..

[14] Swain J., Kumar-Mishra A. (2015). J. Phys. Chem. B..

[15] Ingólfsson H.I., Andersen O.S. (2010). Assay Drug Dev. Technol..

[16] Lundbaek J.A., Birn P., Tape S.E., Toombes G.E., Søgaard R., Koeppe R.E., Gruner S.M., Hansen A.J., Andersen O.S. (2005). Mol. Pharmacol..

[17] Søgaard R., Werge T.M., Bertelsen C., Lundbye C., Madsen K.L., Nielsen C.H., Lundbaek J.A. (2006). Biochem..

[18] Khondker A., Dhaliwal A., Alsop R.J., Tang J., Backholm M., Shi A.C., Rheinstädter M.C. (2017). Phys. Chem. Chem. Phys..

[19] Sierra-Valdez F.J., Forero-Quintero L.S., Zapata-Morin P.A., Costas M., Chavez-Reyes A., Ruiz-Suárez J.C. (2013). PLoS One..

[20] Paloncýová M., Berka K., Otyepka M. (2013). J. Phys. Chem. B..

[21] Maruyama S., Hata T., Matsuki H., Kneshina S. (1997). Biochim. Biophys. Acta..

[22] Hata T., Matsuki H., Kaneshina S. (2000). Biophys. Chem..

[23] Takeda K., Okuno H., Hata T., Nishimoto M., Matsuki H., Kaneshina S. (2009). Colloids Surf. B Biointerf..

